# Microscopic Mechanisms and Pavement Performance of Waterborne Epoxy Resin-Modified Emulsified Asphalt

**DOI:** 10.3390/ma18122825

**Published:** 2025-06-16

**Authors:** Fan Yang, Fang Yu, Hongren Gong, Liming Yang, Qian Zhou, Lihong He, Wanfeng Wei, Qiang Chen

**Affiliations:** 1 Guangxi Beibu Gulf Investment Group Co., Ltd., Nanning 530029, China; 2 National and Local Joint Engineering Research Center for Transportation Civil Engineering Materials, Chongqing Jiaotong University, Chongqing 400074, China; sunnyhlh@cqjtu.edu.cn (L.H.); weiwanfeng@mails.cqjtu.edu.cn (W.W.); 3 School of Road and Bridge Engineering, Guangxi Vocational and Technical College of Communications, Nanning 530023, China; yufangab@163.com; 4 The Key Laboratory of Road and Traffic Engineering, Ministry of Education, Tongji University, Shanghai 201804, China; hgong@tongji.edu.cn; 5 Guangxi Communications Design Group Co., Ltd., Nanning 530029, China; luemingyang@163.com (L.Y.); zhougxjt@163.com (Q.Z.); chenqgxjt@163.com (Q.C.)

**Keywords:** pavement maintenance, waterborne epoxy resin, emulsified asphalt, modification mechanism, microstructure, pavement performance

## Abstract

To address the deficiencies of traditional emulsified asphalt-pavement maintenance material in cohesive strength, high-temperature rutting resistance, as well as adhesion to aggregates, this study developed waterborne epoxy resin-modified emulsified asphalt (WEA) binders using a two-component waterborne epoxy resin (WER) and systematically investigated their modification mechanisms and pavement performance. The results indicated that WER emulsions and curing agents could polymerize to form epoxy resin within the emulsified asphalt dispersion medium, with the modification process dominated by physical interactions. When the WER content exceeded 12%, a continuous modifier network structure was established within the emulsified asphalt. The epoxy resin formed after curing could significantly increase the polarity component of the binder, thereby increasing the surface free energy. The linear viscoelastic range of the WEA binder exhibited a negative correlation with the dosage of the WER modifier. Notably, when the WER content exceeded 6%, the high-temperature stability (rutting resistance and elastic recovery performance) of the binder was significantly enhanced. Concurrently, stress sensitivity and frequency dependence gradually decrease, demonstrating superior thermomechanical stability. Furthermore, WER significantly enhanced the interfacial interaction and adhesion between the binder and aggregates. However, the incorporation of WER adversely affects the low-temperature cracking resistance of the binder, necessitating strict control over its dosage in practical applications.

## 1. Introduction

Asphalt pavements are subjected to the combined effects of environmental factors and traffic loads during their service life, often resulting in early distresses such as anti-skid attenuation, rutting, and cracking. If not treated promptly, it will seriously affect the service life of the pavement and driving safety [1,2,3]. By the end of 2023, the total mileage of China’s expressways had reached 183,600 km, of which over 99% were under maintenance, facing enormous maintenance pressure [4]. The preventive maintenance of road surfaces is the practice of taking maintenance measures on structurally sound road surfaces without increasing their load-bearing capacity to repair road surface defects, extend road surface lifespan, and maintain or improve road surface functionality. Preventive maintenance measures for road surfaces include fog sealing, slurry sealing, thin overlay, micro-surfacing, hot or cold recycling, etc. The above maintenance measures can be categorized based on the types of asphalt, including emulsified asphalt and hot-mix asphalt [5,6,7]. Compared to hot-mix asphalt, emulsified asphalt maintenance materials enable ambient-temperature construction with simplified processes, resulting in significantly reduced energy consumption and volatile organic compound (VOC) emissions. However, traditional polymer-modified emulsified asphalt materials, such as styrene-butadiene rubber (SBR) latex and chloroprene rubber (CR) latex, exhibit significant limitations, including high-temperature sensitivity, poor mechanical properties, and inadequate water stability, which fail to adequately meet the demanding requirements for expressway pavement maintenance [8,9]. Emulsified asphalt modified with waterborne thermosetting resins (such as waterborne polyurethane or waterborne epoxy resin) forms a three-dimensional network structure within the asphalt through polymerization reactions. This modification imparts significant advantages in terms of mechanical properties, rutting resistance, and moisture resistance [10,11]. Consequently, the development of novel, modified, emulsified asphalt materials has emerged as a critical research focus in the road maintenance industry.

Epoxy asphalt material is a high-performance composite formed by incorporating epoxy resin and curing agents into neat asphalt, followed by a curing reaction that creates a three-dimensional cross-linked epoxy network as its skeletal structure. Epoxy asphalt exhibits superior mechanical strength, high-temperature stability, fatigue resistance, and moisture resistance, making it widely applicable in orthotropic steel-deck pavements for long-span bridges [12,13]. Inspired by the performance advantages of epoxy asphalt materials, researchers have proposed the development of waterborne epoxy resin (WER) through the hydrophilization of epoxy resin. This WER is used as a modifier for emulsified asphalt, enabling the formation of high-performance epoxy asphalt materials after water evaporation and modifier curing [14,15,16]. The currently prevalent WER modifier consists of a WER emulsion and a curing agent. When incorporated into emulsified asphalt, this two-component system undergoes polymerization to form a continuous three-dimensional network structure, ultimately enhancing the mechanical, rheological, and durability properties of the binder [17,18,19]. Due to its balanced performance and environmental adaptability, waterborne epoxy resin-modified emulsified asphalt (WEA) has garnered significant attention in the pavement maintenance industry, particularly for applications requiring rapid repair, high adhesion, and long-term service life.

The WEA binder is mainly prepared using a cold mixing method. The WER emulsion and curing agent are mixed into the emulsified asphalt to form high-performance modified asphalt material through a curing reaction and water evaporation [20,21,22]. Yang et al. [23,24] investigated the influence of preparation methods on the performance of WEA binders. They found that a two-step method, which involves first thoroughly mixing the WER emulsion with the curing agent before incorporating it into the emulsified asphalt, could enhance the reaction degree of the WER modifier, resulting in superior performance of the obtained WEA binder. Ji et al. [25,26] studied the effects of different evaporation methods on the properties of WEA binders and found that traditional high-temperature evaporation methods affect the curing reaction of polymers and damage their network structure. Using room-temperature or medium-temperature evaporation methods to prepare WEA binders was recommended. In addition, researchers have also studied the properties of WEA binders and their mixtures. Yan et al. [27] investigated the influence of curing agents on the rheological properties of WEA binders and found that curing agents with a higher number of reactive functional groups promoted greater cross-linking density, leading to improved high-temperature stability of the system. Additionally, the flexibility of the binders was positively correlated with the molecular chain length of the curing agent. Wang et al. [28] prepared AC-13 graded WEA mixtures using WER modifiers with different epoxy values. The study revealed that WEA mixtures with all tested epoxy values exhibited outstanding anti-rutting performance and Marshall stability. However, the WER modifier with an epoxy value of 20 demonstrated the most effective modification performance. He et al. [29,30] found that WER is incompatible with asphalt based on solubility parameters and infrared spectroscopy. The smaller the WER emulsion particles, the more stable the composite structure formed by the WER phase and asphalt phase. A WEA micro-surfacing mixture doped with microwave-activated rubber powder was prepared, which has excellent wear resistance, rutting resistance, and noise reduction performance. Song et al. [31,32] developed a WEA waterproof bonding material and demonstrated that WER can significantly enhance the tensile strength of binders. The optimal ratio of the WEA system for different application scenarios was recommended. Xu et al. [33] developed a WEA cold repair mixture for asphalt-pavement potholes and found that the WER type and content affect the mixture’s construction and pavement performance. The high-temperature stability and moisture stability increase with the increase in WER. Current research efforts have predominantly focused on evaluating the performance of waterborne epoxy-modified emulsified asphalt (WEA) binders and their maintenance materials. However, limited attention has been paid to elucidating the polymerization process of WER modifiers within emulsified asphalt or the underlying interaction mechanisms between WER and asphalt. This current has hindered the further development and widespread application of WEA-based maintenance technologies.

This study employed a two-component non-ionic waterborne epoxy resin (WER) as the primary modifier. A comprehensive investigation was conducted focusing on three key aspects: (1) the polymerization of WER within emulsified asphalt matrices, (2) phase structure evolution in the resulting WEA system, and (3) the modifier’s influence on the surface free-energy characteristics of emulsified asphalt. These systematic analyses ultimately elucidated the fundamental micromechanisms underlying the modification of emulsified asphalt by WER. Furthermore, the modification effects of WER on the pavement performance of emulsified asphalt were quantitatively evaluated through comprehensive characterization, including rheological behavior, low-temperature stability, and adhesion performance. Based on these findings, a WEA binder system tailored for the preventive maintenance of asphalt pavements was developed, demonstrating enhanced durability, thermomechanical stability, and interfacial compatibility for sustainable infrastructure applications.

## 2. Materials and Methods

### 2.1. Raw Material Properties

The WER was a two-component system synthesized in the laboratory. Component A was a non-ionic epoxy emulsion prepared in the laboratory, which was an emulsifier formed by grafting polyether polyol onto E51 epoxy resin and synthesized via the phase inversion method [24]. Component B was a low-viscosity water-soluble curing agent composed of epoxy resin, hydrophilic groups, and triethylenetetramine provided by Chongqing Peimengte Materials Technology Co., Ltd. (Chongqing, China). The basic properties of components A and B are shown in Table 1 and Table 2. The emulsified asphalt selected for this study was a cationic, slow-breaking, neat emulsified asphalt supplied by Qinzhou Luxing Materials Technology Co., Ltd. (Qinzhou, China), featuring a quaternary ammonium salt emulsifier and 70# neat asphalt. The fundamental properties are shown in Table 3.

### 2.2. Preparation of WEA Binder

The WEA binders were synthesized via a two-step method: (1) the WER emulsion and curing agent were precisely weighed in a beaker at a mass ratio of m_A_:m_B_ = 1.5:1 and homogenized manually. To ensure a complete reaction of the WER, the amount of curing agent used at this ratio was approximately 1.2 times the theoretical value. Emulsified asphalt was weighed and placed in a beaker. (2) A predetermined mass of emulsified asphalt was transferred to the beaker, followed by the addition of the WER modifier at dosages of 3%, 6%, 9%, 12%, and 15% (internal mixing method). The mixture was subjected to mechanical stirring at 300–500 rpm for 5 min to ensure uniform dispersion. (3) The resulting WEA mixture was cast into designated molds and cured at ambient temperature for 15 d to facilitate partial water evaporation. Subsequently, the samples were thermally treated in an oven at 80 °C for 12 h to achieve the complete curing of the WEA binders. In addition, the emulsified asphalt residue (0% modifier content) was prepared by evaporating the residue.

### 2.3. Test Methods

#### 2.3.1. Chemical Structure

The attenuated total reflection method (ATR), configured with a Bruker ALPHA II infrared spectrometer (Billerica, MA, USA), was used to analyze the molecular structures of WER modifiers, neat emulsified asphalt, and WEA binders. The specimens were prepared according to the protocol described in Section 2.2. The WEA binder was sectioned into smaller specimens only after complete curing of the modifier and substantial evaporation of residual moisture. The infrared scanning range was 600–4000 cm^−1^, with a scanning frequency of 64 times. Each sample was tested three times.

#### 2.3.2. Micromorphology

The surface micromorphology of the cured WEA binder was observed using the OLYMPUS-BX41 fluorescence microscope (Tokyo, Japan). We took 0.5–1.5 g of WEA mixture and applied it onto a glass slides with lengths, widths, and thicknesses of 76 mm, 26 mm, and 1.5 mm, respectively. After standard curing (outlined in Section 2.2), we conducted a microstructure test. The magnification was 100 times.

#### 2.3.3. Surface Free Energy (SFE)

The contact angle test was used to indirectly analyze the variation law of surface free energy (SFE) of the WEA binder, and the preparation process was consistent with Section 2.2.

**Figure 1:**
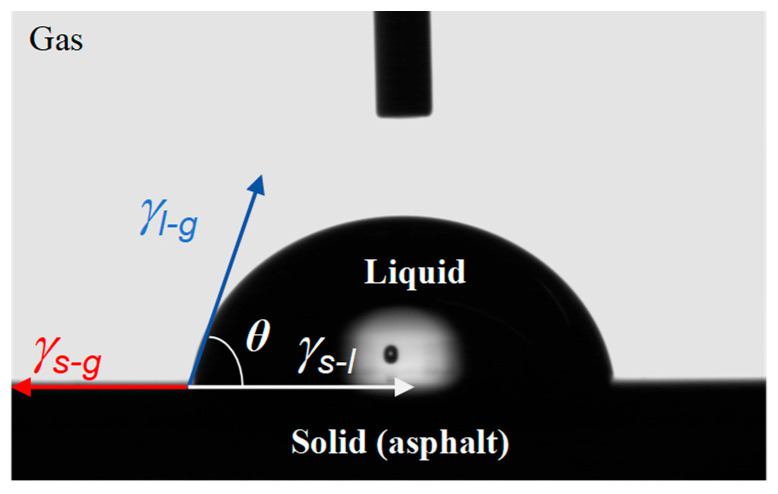
The infiltration process of liquid on asphalt surface.

Due to the solid or semi-solid state of asphalt materials at room temperature, it is not easy to measure their SFE directly. Fowkes [34] and Good [35] proposed the sessile drop method for indirectly measuring SFE. Figure 1 shows the wetting process of a liquid on a surface when a liquid with a known SFE is dropped onto the surface of asphalt material. According to Young’s theory, the mechanical equation in equilibrium can be obtained, as shown in Equation (1):

(1) $$\gamma_{s-g}=\gamma_{l-g}\cos\theta +\gamma_{s-g}$$

where *γ_s-l_*, *γ_s-g_* and *γ_l-g_* are the liquid–solid, solid–gas, and liquid–gas interface tension, respectively, mJ/m^2^; and *θ* is the contact angle between liquid and solid.

The SFE of a material is composed of dispersion and polarity components, and its calculation process is shown in Equations (2) and (3).

(2) $$\gamma_{l}=\gamma_{l}^{LW}+\gamma_{l}^{AB}$$

(3) $$\gamma_{s}=\gamma_{s}^{LW}+\gamma_{s}^{AB}$$

where *γ_l_* and *γ_s_* represent the SFE of liquid and solid, respectively, mJ/m^2^; $\gamma_{l}^{LW}$ and $\gamma_{s}^{LW}$ denote the dispersion components of the SFE for the liquid and solid, respectively, mJ/m^2^; and $\gamma_{l}^{AB}$ and $\gamma_{s}^{AB}$ represent the polar components of the SFE for the liquid and solid, respectively, mJ/m^2^.

Owens et al. proposed that the SFE of the liquid–solid interface can be calculated by the geometric mean of the dispersion and polarity components of liquids and solids [36]. The liquid–solid SFE can be expressed as Equation (4):

(4) $$\gamma_{s-l}=\gamma_{s}+\gamma_{l}-2\sqrt{\gamma_{s}^{LW}\gamma_{l}^{LW}}-2\sqrt{\gamma_{s}^{AB}\gamma_{l}^{AB}}$$

Combining Equations (1)–(4), the dispersion and polar components of the SFE of asphalt material can be obtained, as shown in Equation (5):

(5) $$\frac{1+\cos\theta }{2}\frac{\gamma_{l}}{\sqrt{\gamma_{l}^{LW}}}=\sqrt{\gamma_{s}^{AB}}\times \sqrt{\frac{\gamma_{l}^{AB}}{\gamma_{l}^{LW}}}+\sqrt{\gamma_{s}^{LW}}$$

According to Equation (5), when three or more liquid SFE parameters are known, linear fitting is performed with $\sqrt{\gamma_{l}^{AB}/\gamma_{l}^{LW}}$ as the independent variable and $\gamma_{l}(1+\cos\theta )/2\sqrt{\gamma_{l}^{LW}}$ as the dependent variable. The square of the slope in the fitting result is the polarity component of the asphalt material SFE, and the square of the intercept is the dispersion component. Distilled water, glycerol, and formamide were selected as the test liquids for the WEA binder, and their SFE parameters are shown in Table 4.

#### 2.3.4. High-Temperature Rheological Properties

The high-temperature rheological properties of the WEA binder under different WER dosages were analyzed using TA company’s HR-10 dynamic shear rheometer (DSR) (Boston, MA, USA), including linear viscoelasticity range, temperature dependence, frequency dependence, and creep and recovery. Due to the high modulus of the WEA binder under a high modifier dosage, 25 mm parallel plates were used when the WER modifier dosage did not exceed 6%, and 8 mm parallel plates were used when the WER modifier dosage exceeded 6%. Each sample was tested twice. For rheological samples, the mixed WEA binder was poured into silicone molds with dimensions of either Φ25 mm × 1 mm or Φ8 mm × 2 mm. The rheological properties were then tested after curing, as described in Section 2.2.

The influence of the WER on the linear viscoelastic behavior of WEA binders was evaluated using strain sweep mode with a strain range of 0.1–100% at test temperatures of 35 °C and 50 °C. In accordance with ASTM D7175 [37], the strain level at which the complex modulus decreases to 90% of its initial value was defined as the upper limit of the linear viscoelastic range.

Following ASTM D6373 [38], the temperature-dependent rheological properties of the WEA binder, including the rutting factor, storage modulus, loss modulus, and phase angle, were evaluated using DSR temperature sweep tests. The temperature range was set from 52 °C to 88 °C with an incremental step of 6 °C.

Frequency sweep tests were conducted under DSR oscillation mode to investigate the frequency-dependent viscoelastic behavior of the WEA binder at high service temperature (64 °C). The complex modulus and phase angle were measured over an angular frequency range of 0.1–100 rad/s to assess the binder’s response under varying loading frequencies.

The high-temperature elastic recovery performance of the WEA binder was evaluated using the Multiple Stress Creep and Recovery (MSCR) tests at two stress levels—0.1 kPa (low stress) and 3.2 kPa (high stress)—with a constant test temperature of 64 °C. By ASTM D7405 [39], after ten cycles of creep recovery of the WEA binder, the elastic recovery rate *ε_r_*_(*τ*,*N*)_ and unrecoverable creep compliance *J_nr_*_(*τ*,*N*)_ under every cycle were calculated as shown in Equations (6) and (7):

(6) $$\varepsilon_{r}(\tau ,N)=\frac{\left(\varepsilon_{1}-\varepsilon_{10}\right)}{\varepsilon_{1}}\times 100$$

(7) $$J_{nr}(\tau ,N)=\frac{\varepsilon_{10}}{\tau }$$

where *ε*_1_ and *ε*_10_ represent the strain values at the end of each cycle of creep and elastic recovery, respectively.

The average elastic recovery rate (*R*) and unrecoverable creep compliance (*J_nr_*) under ten creep-recovery cycles were calculated as shown in Equations (8) and (9):

(8) $$R=\frac{\sum_{N=1}^{10}SUM(\varepsilon_{r}(\varepsilon ,N))}{10}$$

(9) $$J_{nr}=\frac{\sum_{N=1}^{10}SUM(J_{nr}(\varepsilon ,N))}{10}$$

Generally, the stress sensitivity of asphalt materials under different stresses (0.1 kPa and 3.2 kPa) is evaluated by *J_nrdiff_*, and the calculation formula is shown in Equation (10):

(10) $$J_{nrdiff}=\frac{\left(J_{nr,3.2}-J_{nr,0.1}\right)}{J_{nr,0.1}}$$

However, Stempihar et al. [40] demonstrated that the *J_nrdiff_* parameter fails to accurately characterize the stress sensitivity of asphalt binders with low *J_nr_*_,3.2_ values. Their research revealed that a newly proposed parameter, *J_nrslope_*, provides a more reliable assessment of binder stress sensitivity and enables the more precise prediction of rutting resistance performance [41]. The calculation methodology is presented in Equation (11):

(11) $$J_{nrslope}=\frac{dJ_{nr}}{d\tau }=\frac{\left|J_{nr,3.2}-J_{nr,0.1}\right|}{3.1}\times 100$$

Compared to Equation (10), the denominator of Equation (11) does not contain *J_nr_*_,0.1_, but rather the difference in stress. *J_nrslope_* is more suitable for evaluating the stress sensitivity of asphalt materials under different stress levels.

#### 2.3.5. Low-Temperature Creep Performance

The Bending Beam Rheometer (BBR) was used to analyze the low-temperature creep characteristics of the WEA binder and to evaluate the low-temperature crack resistance of various types of asphalt using the creep stiffness modulus and creep rate as indicators. The coolant used in the experiment was anhydrous ethanol, which did not affect the testing of the sample. The test temperatures were −12 °C and −18 °C. Due to the curing process required for the WEA binder in this article, traditional BBR molds could not meet the usage requirements. Therefore, silicone molds were selected for sample preparation, with groove lengths, widths, and depths of 127 mm, 12.7 mm, and 6.4 mm. After undergoing standard curing (as outlined in Section 2.2), the excess surface of the sample was scraped off. The relationship curve between the creep stiffness modulus and time was recorded during the experiment. Three samples were tested for each WEA binder.

#### 2.3.6. Mechanical Properties

According to ASTM D4541 [42], the pull-off strength was tested using the PosiTest ATA20A-B adhesion tester produced by DeFelsko (New York, NY, USA) to evaluate the adhesion between the binder and aggregate. A 20 mm diameter stainless pull-off stub was used for testing. The aggregates were made of basalt material with dimensions of 100 mm × 100 mm × 10 mm and a polished value of 1000. A 1–2 g sample of the composite binder mixture was deposited within a silicone ring (22 mm inner diameter). The specimens were cured following the conditioning procedure detailed in Section 2.2. After complete moisture evaporation, a conventional two-component epoxy resin adhesive (commercially available Type A/B) was uniformly applied to the WEA binder surface. The pull-off stub was then immediately pressed onto the specimen surface with firm contact. The assemblies were maintained at ambient temperature (23 ± 2 °C) for a minimum of 12 h to ensure complete epoxy resin curing prior to testing. All tests were conducted at a controlled loading rate of 0.7 MPa/s. The stress–strain curves and the maximum pull-off strength at fracture were recorded. For statistical reliability, five replicate specimens were prepared and tested for each asphalt material formulation.

**Figure 2:**
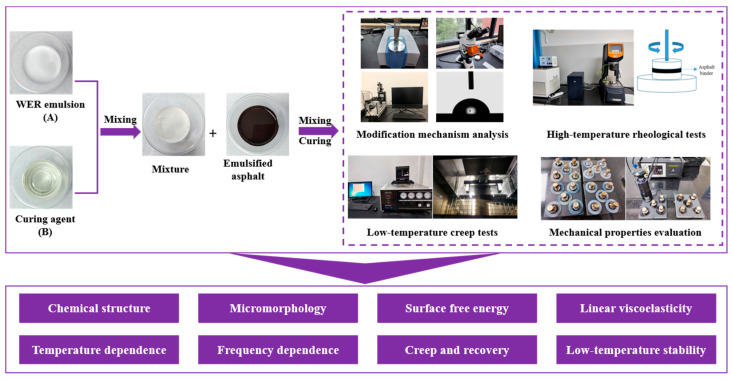
Flowchart of experiment design.

This study investigated the polymerization process and modification mechanisms of WER modifiers within emulsified asphalt while systematically analyzing their impact on the macroscopic properties of the resulting binders. The experimental design flowchart is shown in Figure 2.

## 3. Results

### 3.1. Modification Mechanism

#### 3.1.1. Chemical Structural Changes

**Figure 3:**
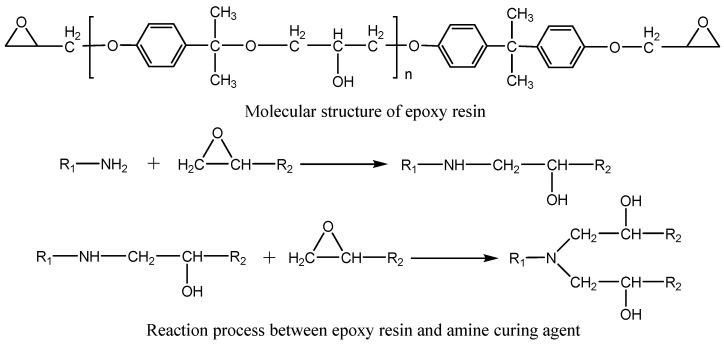
E51 epoxy resin and its reaction process with curing agent.

**Figure 4:**
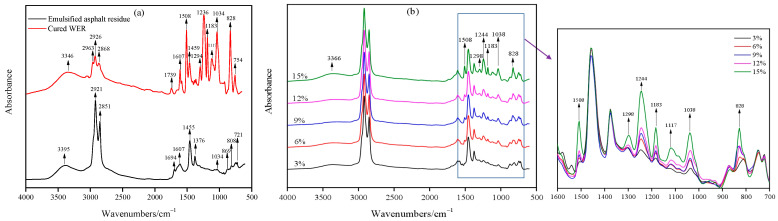
Infrared spectra: (**a**) WER modifier and emulsified asphalt residue, (**b**) WEA binders.

The molecular structure changes of the WEA binder, cured WER modifier, and emulsified asphalt residue were analyzed through ATR-FTIR. Among them, the WER emulsion prepared includes E51 epoxy resin as the effective component and a modified amine curing agent as the curing agent. Amine curing agents are commonly used to cure waterborne epoxy resins. These curing agents prepare coatings or materials with high chemical resistance after curing, which can resist the corrosion of acids, alkalis, oils, and some solvents [43,44]. Meanwhile, the curing time, hardening speed, and final performance can be adjusted according to specific application requirements. Amine curing agents have strong adaptability and broad applicability, and their reaction process with E51 epoxy resin is shown in Figure 3. The infrared spectra are shown in Figure 4.

The characteristic peaks of raw materials and the WEA binder are listed in Table 5. The absorption peaks of emulsified asphalt residue indicate that its infrared spectrum primarily includes alkyl and aromatic hydrocarbon peaks, as well as peaks corresponding to -NH (3346 cm^−1^) and C-O (1034 cm^−1^) derivatives, which originate from the cationic emulsifier [24]. As shown in Figure 4a, the cured WER molecular structure contains numerous polar functional groups. For instance, the peak at 3346 cm^−1^ corresponds to hydroxyl compounds formed by the reaction between epoxy groups and amine groups, the peak at 1739 cm^−1^ represents the C=O bond, and the peaks at 1034–1298 cm^−1^ are attributed to C-O bonds. Notably, the absence of an absorption peak at 910 cm^−1^ in the cured WEA indicates the complete consumption of epoxy groups during the reaction. The infrared spectrum of cured WER is consistent with the reaction results shown in Figure 3. From Figure 4b, it is evident that as the modifier dosage increases, the intensity of epoxy resin absorption peaks in the WEA binder within the range of 828–1508 cm^−1^ gradually increases, adhering to the Lambert–Beer law, where peak intensity is positively correlated with functional group concentration. No additional absorption peaks were observed in the WEA binder spectrum beyond those presented in Figure 4a. This spectral evidence suggests that the modification mechanism of emulsified asphalt by the WER primarily involves physical interactions rather than the formation of chemical bonds.

#### 3.1.2. Micromorphology Changes

**Figure 5:**
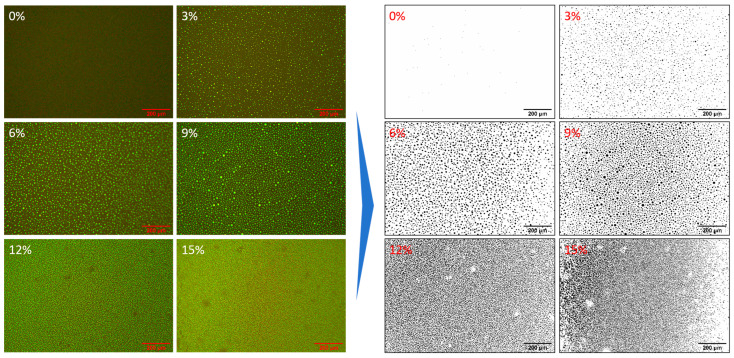
The distribution of asphalt phase and WER phase in the binder system under different modifier content (100×).

From the molecular polarity perspective, the WER modifier’s molecular structure contains more N and O atoms, causing the electron clouds of nearby C, H, and other atoms to shift towards N and O atoms. The final result is that atoms with strong electronegativity carry negative charges, while those with weak electronegativity carry positive charges. Therefore, the WER modifier in this article belongs to strongly polar materials, whereas the emulsified asphalt, primarily composed of hydrocarbons, is characterized as a weakly polar material. Theoretically, WER and emulsified asphalt have significant polarity differences, making it challenging to achieve molecular-level blending and inevitably resulting in a two-phase structure [30,31]. Fluorescence microscopy was used to observe the microstructural surface morphology of WEA binders with modifier dosages ranging from 0% to 15%, with a magnification of 100 times. ImageJ 1.53k software was used for black-and-white processing and particle size analysis of fluorescent patterns. The results are presented in Figure 5.

When the modifier dosage is 0%, almost no fluorescence reaction is observed in the emulsified asphalt residue. This is because the emulsified asphalt residue primarily consists of hydrocarbons, and the activation energy of C-H bonds is relatively high. It is difficult to convert hydrocarbons into excited states when ultraviolet light is irradiated on the surface of the residue, making it challenging to observe fluorescence images. A significant fluorescence reaction occurred in the system when the WER modifier was added. The reason is that many polar groups in the modifier produce fluorescent reactions when exposed to ultraviolet radiation [45]. From the fluorescence images, it can be observed that both the WER modifier and emulsified asphalt, being O/W (oil-in-water) systems, can be easily and uniformly mixed through simple blending. With the increase in modifier dosage, WER particles gradually tend to aggregate, and the particle size gradually increases. The average particle sizes of WER at 3%, 6%, and 9% dosages are 11.7 μm, 25.7 μm, and 28.8 μm, respectively. When the modifier dosage exceeds 12%, the WER gradually forms a continuous phase within the emulsified asphalt. From Figure 5, it can also be seen that WER can be uniformly dispersed as particles in asphalt, indicating good compatibility between the WER system and emulsified asphalt. The reason is that the WER system involved is non-ionic, compatible with various emulsified asphalts, and does not affect the charge balance and stability of the emulsified asphalt.

#### 3.1.3. Evolution Process of the SFE

**Figure 6:**
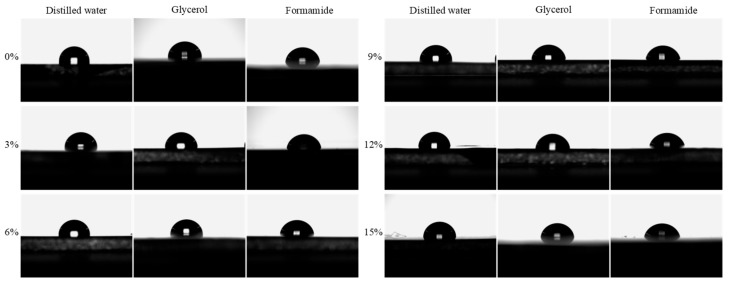
Contact angle of WEA binders.

**Figure 7:**
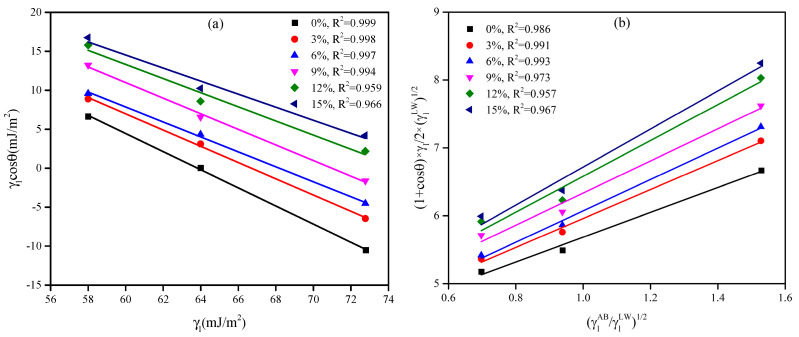
The process of determining SFE: (**a**) data validation; (**b**) linear fitting of SFE parameters.

The surface free energy (SFE) of asphalt materials serves as a fundamental indicator for evaluating both cohesive strength within the binder matrix and adhesive performance at the asphalt–aggregate interface. Thermodynamically, higher SFE values promote superior interfacial bonding, thereby creating a more robust moisture-resistant system. The influence of the WER modifier on the SFE properties of the binder system was quantitatively assessed using the contact angle method (ASTM D7490) [46]. The experimental results are shown in Figure 6 and Table 6. Distilled water, glycerol, and formamide, all of which are polar liquids, were used as test liquids. As the modifier dosage increased, the contact angles between the WEA binder and the three liquids gradually decreased. This indirectly demonstrates that the modifier enhances the polarity of the binder system. The validity of the contact angle data was verified [47], as shown in Figure 7a. A significant linear relationship was observed between the SFE (*γ*) of the three liquids and *γcosθ*, confirming the reliability of the test data.

**Figure 8:**
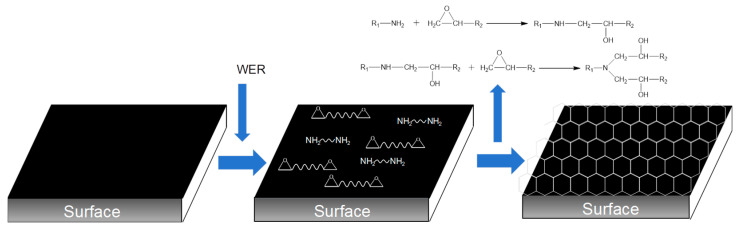
Surface modification process of WER on asphalt binder.

According to the formula for calculating SFE in Section 2.3.3, linear fitting was performed on the contact angle data, and the final experimental results are presented in Figure 7b and Table 7. In the emulsified asphalt residue (0% sample), the dispersion component, which represents the non-polar fraction, is significantly higher than the polar component. This is because the emulsified asphalt residue primarily consists of neat asphalt, which is mainly composed of hydrocarbons with minimal polar functional groups. As a result, the intermolecular forces are dominated by van der Waals forces [48]. After adding the WER modifier, the SFE of the system gradually increased. The SFE of the binder increased by 5.7%, 7.2%, 17.7%, 23.9%, and 27.9% at 3%, 6%, 9%, 12%, and 15% modifier content, respectively. From the perspective of the SFE component, as the WER content increases, the dispersion component of the binder remains almost unchanged. In contrast, the polarity component gradually increases, ultimately increasing the total surface free energy. The addition of the WER has the most significant impact on the polarity component. The polarity component of the binder increases by 35.5%, 59.4%, 67.8%, 108.1%, and 134% at 3%, 6%, 9%, 12%, and 15% modifier content, respectively. The polar component of the binder exhibited the most pronounced enhancement when the modifier content exceeded 12%. This phenomenon can be attributed to the intrinsically polar nature of the WER modifier. The surface modification process of asphalt by WER is illustrated in Figure 8. The reaction between the WER emulsion and curing agent generates a substantial number of nitrogen-containing and hydroxyl-containing compounds, which collectively enhance the overall polarity of the binder system. This chemical transformation directly contributes to the increased polar component of the system’s SFE.

### 3.2. High-Temperature Rheological Properties

#### 3.2.1. Linear Viscoelasticity

**Figure 9:**
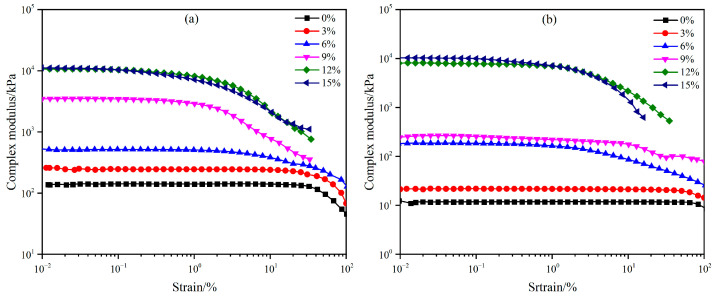
Strain scanning results of WEA binder at different temperatures: (**a**) 35 °C; (**b**) 50 °C.

The linear viscoelasticity of asphalt refers to the characteristic of asphalt materials exhibiting both viscous and elastic behaviors within a small strain range. This property enables asphalt to store energy and recover deformation like an elastic solid when subjected to external forces, while simultaneously dissipating energy and undergoing irreversible flow like a viscous fluid. The linear viscoelastic range of WEA binders with modifier contents ranging from 0% to 15% was determined through strain sweep tests conducted following ASTM D7175. The tests were conducted at temperatures of 35 °C and 50 °C, and the results are presented in Figure 9.

The complex modulus of the six WEA binders decreases with increasing temperature. When the modifier content is below 12%, the complex modulus of the binders decreases rapidly, whereas the influence of temperature on WEA binders with modifier concentrations of 12% and 15% is relatively minor. Additionally, at the same temperature, the linear viscoelastic range of the binders gradually narrows with increasing modifier content. At 35 °C, the linear viscoelastic ranges of WEA binders with 0%, 3%, 6%, 9%, 12%, and 15% modifier content are 31.91%, 15.92%, 0.98%, 0.51%, 0.26%, and 0.13%, respectively. At 50 °C, the linear viscoelastic ranges of WEA binders with 0%, 3%, 6%, 9%, 12%, and 15% modifier content are 65.21%, 50.49%, 1.02%, 0.97%, 0.65%, and 0.20%, respectively. It is evident that, at both temperatures, when the WER content reaches 6% or higher, the linear viscoelastic range of the binders decreases significantly. This phenomenon can be attributed to the fact that at lower modifier concentrations, the asphalt phase dominates the WEA system, resulting in rheological behavior characteristic of conventional asphalt materials. As the modifier content increases, the proportion of WER emulsion and curing agent in the system rises, leading to the gradual aggregation of the synthesized epoxy resin within the emulsified asphalt to form a continuous network. This network increasingly contributes to the performance of the binder system. Consequently, the flow properties of the binder are reduced, and it exhibits characteristics of an elastic material. As shown in Figure 9, when the WER content exceeds 12%, the influence of temperature on the complex modulus of the binder becomes increasingly negligible. For the convenience of subsequent performance comparison, all rheological tests of the WEA binder in this article were conducted using a strain of 0.1%.

#### 3.2.2. Temperature Dependence

**Figure 10:**
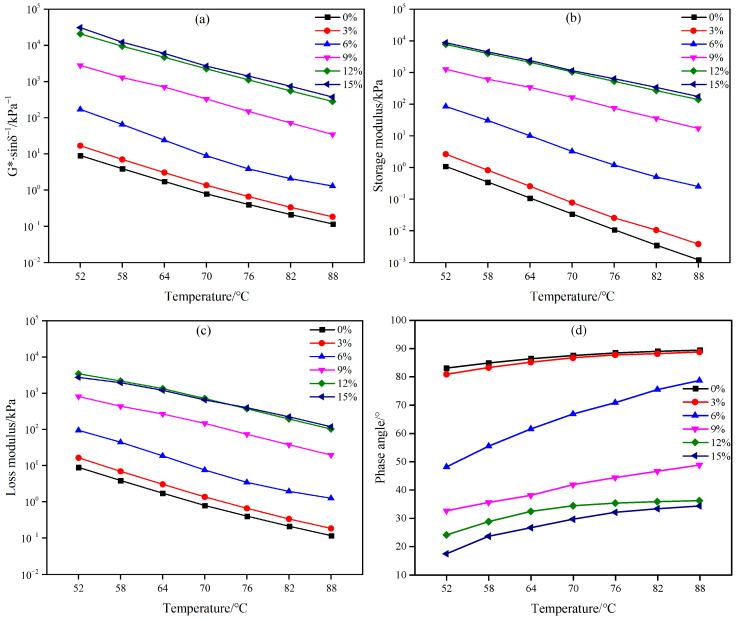
WEA binder temperature dependence: (**a**) rutting factor; (**b**) storage modulus; (**c**) loss modulus; (**d**) phase angle.

The strength formation process of emulsified asphalt significantly differs from that of hot-mix asphalt. Hot-mix asphalt possesses a highly uniform colloidal structure, whereas the strength development of emulsified asphalt involves processes such as demulsification, water evaporation, and coalescence of asphalt particles [2]. As a result, traditional emulsified asphalt binders generally exhibit inferior high-temperature stability compared to hot-mix asphalt. The high-temperature rheological properties of WEA binders were investigated using temperature sweep tests conducted with a DSR. The experimental results are presented in Figure 10.

As illustrated in Figure 10a, the rutting factor of WEA binders exhibits a positive correlation with modifier content. When the modifier content is below 6%, the asphalt binder remains the dominant phase in the WEA system, resulting in a limited improvement in rutting resistance. As the modifier content reaches 6–12%, the binder system becomes increasingly populated with modifier particles that tend to aggregate and form network-like structures, leading to a significant enhancement in rutting resistance. When the modifier content exceeds 12%, a continuous network structure is established within the binder, and the contribution of the WER to deformation resistance becomes more pronounced. Consequently, the temperature sensitivity of the rutting factor in the WEA system gradually diminishes [27,32]. Figure 10b,c demonstrate that both the loss modulus and storage modulus of WEA binders within the 0–15% modifier range decrease with increasing temperature. This behavior is attributed to the fact that the system still contains over 85% asphalt. At high temperatures, the asphalt phase undergoes softening, maintaining the viscoelastic performance of the WEA binders. This softening effect directly reduces the storage modulus and loss modulus of the binders.

The phase angle further elucidates the high-temperature viscoelastic characteristics of WEA binders. As depicted in Figure 10d, the phase angle of WEA binders with 0% and 3% modifier content approaches 90° at high temperatures, indicating that the system tends to flow, and the modifier contributes minimally to improving deformation resistance. When the modifier content reaches 6% or higher, the phase angle of WEA binders decreases significantly. Notably, for binder formulations exceeding 12% modifier content, the temperature-dependent variation in phase angle diminishes considerably. Above 76 °C, the phase angle stabilizes, suggesting that the deformation resistance of the system becomes predominantly governed by the WER. These results demonstrate that WER effectively enhances the high-temperature rutting resistance of emulsified asphalt. However, the high-temperature stability of the binders exhibits a strong dependency on modifier content. Specifically, higher modifier concentrations (≥12%) not only suppress temperature sensitivity but also establish a modifier-dominated structural framework, thereby substantially improving the thermomechanical stability of the system.

#### 3.2.3. Frequency Dependence

**Figure 11:**
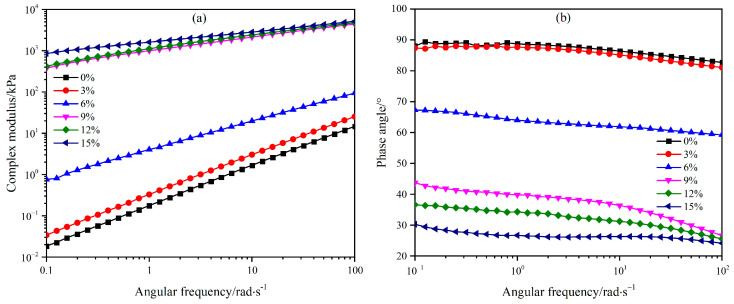
Frequency scanning results of WEA binder: (**a**) complex modulus; (**b**) phase angle.

Asphalt binders exhibit non-Newtonian fluid behavior at high temperatures, where their performance becomes more susceptible to the effects of loading frequency. Specifically, the modulus increases under high-frequency loading but decreases under low-frequency conditions. This phenomenon explains why high-temperature environments, such as bus stops and intersections subjected to slow-speed traffic, are more prone to rutting deformation [49,50]. To evaluate the frequency-dependent behavior of WEA binders, frequency sweep tests were conducted using a DSR at 64 °C. The complex modulus and phase angle of the binders were analyzed, and the results are presented in Figure 11.

Figure 11a demonstrates that WEA binders exhibit significant non-Newtonian fluid characteristics under high-temperature conditions, where the modulus of the system gradually increases with rising frequency. Notably, when the WER content is below 6%, the complex modulus of the binders shows strong frequency dependence, with orders-of-magnitude changes in modulus, reflecting the typical behavior of unmodified asphalt materials. However, once the modifier content exceeds 6%, the complex modulus of the binders increases substantially, and its sensitivity to frequency diminishes. As shown in Figure 11b, the phase angles of all six WEA binder formulations decrease with increasing frequency. For binders with 0% and 3% modifier content, the phase angle approaches 90° at elevated temperatures, indicating dominant viscous behavior characteristic of asphalt materials, which results in pronounced frequency dependence. In contrast, higher modifier contents (≥6%) reduce both the phase angle magnitude and its temperature sensitivity. These results highlight that the frequency-dependent behavior of WEA binders is intrinsically linked to modifier content. Higher WER dosages enhance the modulus and elastic contribution of the system, thereby reducing frequency dependence and improving high-temperature rutting resistance. This phenomenon can be attributed to the formation of a modifier-dominated network structure at elevated WER contents, which stabilizes the rheological response under varying loading frequencies and suppresses the viscous flow behavior of asphalt.

#### 3.2.4. Creep and Recovery

**Figure 12:**
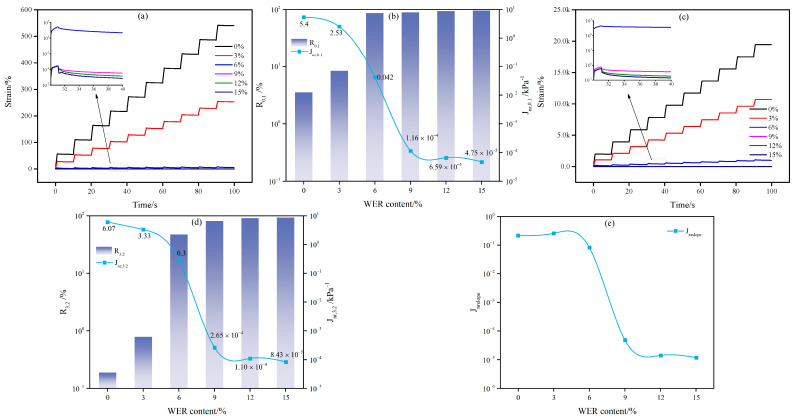
MSCR test results of WEA binder: (**a**) creep and recovery curve (0.1 kPa); (**b**) *R*_0.1_ and *J_nr_*_,0.1_; (**c**) creep and recovery curve (3.2 kPa); (**d**) *R*_3.2_ and *J_nr_*_,3.2_, (**e**) *J_nrslope_*.

The high-temperature elastic recovery performance of WEA binders was evaluated using the Multiple Stress Creep Recovery (MSCR) test. The strain curves obtained from 10 creep-recovery cycles are illustrated in Figure 12. The average values of the elastic recovery rate (*R*) and non-recoverable creep compliance (*J_n_*_r_) over the ten cycles were calculated to quantify the viscoelastic response and permanent deformation resistance of the binders.

As shown in Figure 12a, significant differences are observed in the strain curves of WEA binders under a 0.1 kPa stress at high temperatures. The strain values after a single creep-recovery cycle for binders with 0% and 3% modifier content are substantially higher than those of other binders. Furthermore, the strain response of WEA binders progressively decreases with increasing modifier content. The elastic recovery rates (*R*_0.1_) for WEA binders with 0%, 3%, 6%, 9%, 12%, and 15% modifier content are 3.54%, 8.44%, 87.46%, 90.73%, 94.94%, and 96.64%, respectively. A critical threshold is identified at 6% modifier content; below this value, the WEA binders exhibit negligible elastic recovery (R0.1 < 10%), whereas formulations with ≥6% modifier content demonstrate a dramatic improvement in elastic recovery, achieving R0.1 > 85%. As is evident from Figure 12b, the *J_nr_*_,0.1_ value decreases dramatically when the modifier content exceeds 6%. With the modifier content increasing from 0% to 15%, the *J_nr_*_,0.1_ of the WEA binder exhibits a significant reduction from 5.4 kPa^−1^ to 4.75 × 10^−5^ kPa^−1^. These results confirm that after 6% modifier content, the WEA system contains sufficient WER to establish a modifier-dominated framework, significantly enhancing the high-temperature elastic recovery and reducing permanent deformation susceptibility.

At a stress level of 3.2 kPa, the creep-recovery curves exhibit trends consistent with those observed at 0.1 kPa, as illustrated in Figure 12c and d. The elastic recovery rates (*R*_3.2_) for WEA binders with 0%, 3%, 6%, 9%, 12%, and 15% modifier content are 0.19%, 0.79%, 47.65%, 81.99%, 91.85%, and 94.22%, respectively. Under high-stress conditions and with low modifier content (<6%), the elastic recovery ratio of the WEA binder approaches negligible levels (*R*_3.2_ ≈ 0). However, when the modifier content exceeds 6%, the high-temperature elastic recovery performance improves significantly, with the recovery ratio showing minimal dependence on stress levels. For instance, even at 3.2 kPa stress, formulations with modifier content ≥ 12% maintain elastic recovery rates exceeding 90%. Concurrently, as the modifier content increases from 0% to 15%, the *J_nr_*_,3.2_ value of the WEA binder decreases substantially from 6.07 kPa^−1^ to 8.43 × 10^−5^ kPa^−1^. According to ASTM D8239 [51], binders with *J_nr_*_,3.2_ values below 0.5 kPa^−1^ qualify for use in extremely heavy traffic conditions. Consequently, WEA binders with modifier content exceeding 6% meet this criterion, demonstrating suitability for high-stress applications. These results conclusively validate that WER significantly enhances the high-temperature rutting resistance of emulsified asphalt binders by reinforcing their elastic recovery capacity and reducing permanent strain accumulation.

The influence of stress levels on the high-temperature elastic performance of WEA binders was evaluated using the Jnrslope parameter, as illustrated in Figure 12e. There is a significant negative correlation between Jnrslope and WER content. Notably, when the modifier content exceeded 6%, the effect of stress on the non-recoverable creep compliance of WEA binders decreased significantly, indicating reduced stress sensitivity. This trend suggests that higher WER dosages enhance the structural stability of the binder system, effectively mitigating stress-dependent permanent deformation under elevated temperature conditions.

### 3.3. Low-Temperature Stability

**Figure 13:**
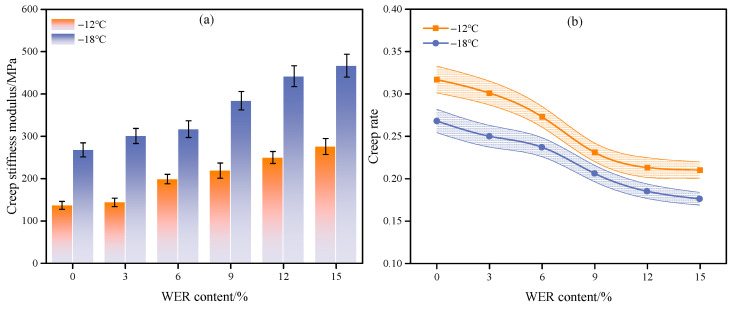
BBR test results: (**a**) creep stiffness modulus; (**b**) creep rate.

The low-temperature stability of WEA binders with modifier contents ranging from 0% to 15% was evaluated using the BBR tests at −12 °C and −18 °C. The test results, including creep stiffness and creep rate, are presented in Figure 13.

As shown in Figure 13a, the creep stiffness modulus of the WEA binder gradually increases with decreasing temperature. This phenomenon occurs because the WEA binder contains a significant asphalt fraction, whose mechanical properties are highly temperature-dependent. At lower temperatures, lighter fractions within the asphalt structure—such as saturates, aromatics, and resins—solidify, restricting the extensibility of molecular chains and resulting in a significant increase in modulus. Furthermore, at identical temperatures, the creep stiffness modulus of the WEA binder increases progressively with higher WER modifier content. When the WER content is below 6%, the modifier exhibits minimal influence on the low-temperature performance of emulsified asphalt, primarily because the asphalt phase remains dominant in the binder system, thereby preserving its inherent low-temperature characteristics. However, once the WER content reaches or exceeds 6%, the stiffness modulus of the binders increases significantly. This phenomenon is attributed to the high cross-linking density and strength of the WER modifier, coupled with its elevated glass transition temperature. At low temperatures, the spatial hindrance effect of WER restricts the molecular chain mobility of the binder system, reducing flexibility and enhancing rigidity [52]. As the modifier content increases, the epoxy resin generated through the polymerization of the WER emulsion and the curing agent gradually aggregates within the emulsified asphalt, forming a three-dimensional network structure. Concurrently, the contribution of epoxy resin to the system becomes increasingly pronounced, resulting in enhanced stiffness modulus. As shown in Figure 13b, the lower the temperature, the lower the creep rate of the WEA binder. Moreover, the creep rate of the binder decreases with increasing modifier content. This reduction occurs because WER enhances the overall stiffness and hardness of the binder, leading to slower creep responses under constant stress. Therefore, WER modifiers have adverse effects on the low-temperature toughness of WEA binders.

### 3.4. Mechanical Properties

**Figure 14:**
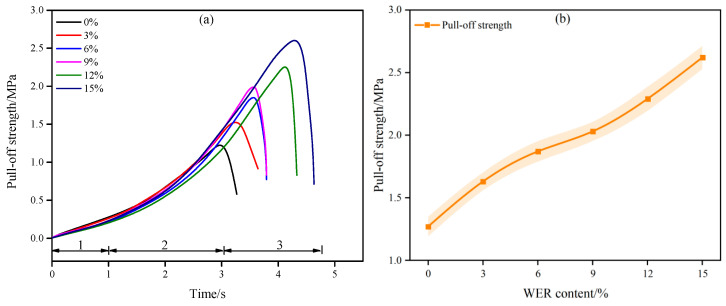
Results of adhesion test for WEA binder: (**a**) pull-off curves; (**b**) pull-off strength.

The bonding mechanism between emulsified asphalt and aggregates differs significantly from that of hot-mix asphalt, rendering emulsified asphalt maintenance materials more susceptible to moisture-induced damage. The adhesion strength between the binder and aggregates directly governs the moisture stability of emulsified asphalt-based maintenance systems, where stronger interfacial adhesion correlates with enhanced resistance to water degradation. In accordance with ASTM D4541, the adhesion properties of WEA binders to aggregates were evaluated through pull-off strength testing, with the results presented in Figure 14.

Under controlled stress loading rates, the pull-off curves of six WEA binder formulations exhibit significant similarity, as shown in Figure 14a. The pull-off curves can be divided into three distinct stages. Elastic stage: The tensile stress increases linearly with time, corresponding to disordered molecular chain arrangements in the binder system. Yield stage: The tensile stress rises sharply with time as molecular chains gradually align and tighten along the loading direction. Failure stage: The material reaches its ultimate tensile state and undergoes structural failure [41]. As clearly illustrated in Figure 14b, the pull-off strength of the binders increases significantly with higher WER modifier content. The pull-off strengths for WEA binders with 0%, 3%, 6%, 9%, 12%, and 15% modifier content are 1.27 MPa, 1.63 MPa, 1.81 MPa, 2.03 MPa, 2.29 MPa, and 2.62 MPa, respectively. Compared to the 0% modifier formulation, the pull-off strengths of binders with 3%, 6%, 9%, 12%, and 15% WER content increase by 28.34%, 47.24%, 59.84%, 80.31%, and 106.30%, respectively. This enhancement is attributed to the inherent high strength of the WER and its polar nature, which strengthens the interfacial adhesion between the binder and aggregates, thereby improving pull-off resistance. The polar functional groups in WER promote chemical interactions and physical bonding at the binder–aggregate interface, effectively mitigating moisture-induced debonding and enhancing long-term durability.

## 4. Conclusions and Recommendations

This study prepared a waterborne epoxy resin-modified emulsified asphalt (WEA) binder and investigated its microscopic modification mechanism and pavement performance. The main findings are summarized as follows:

(1) The modification process of the WER on emulsified asphalt involves both the chemical synthesis of epoxy resin and physical modification. The average particle sizes of WER at 3%, 6%, and 9% dosages are 11.7 μm, 25.7 μm, and 28.8 μm, respectively. However, when the dosage reaches 12% or higher, WER forms a continuous network structure within the system.
(2) The incorporation of WER significantly affects the SFE of emulsified asphalt. At modifier contents ranging from 3% to 15%, the SFE of the binder increases by 5.7% to 27.9%, with the polar component rising by 35.5% to 134%, while the non-polar component remains nearly unaffected. This enhancement in SFE improves the interfacial compatibility and adhesion properties of the binder.
(3) Under WER dosages of 0–15%, the linear viscoelastic range of the binder at 35 °C and 50 °C varies between 31.91–0.13% and 65.21–0.2%, respectively. When the modifier content exceeds 6%, the binder exhibits significant improvements in high-temperature rutting resistance and elastic recovery, along with reduced stress sensitivity and frequency dependence.
(4) WER enhances the mechanical strength and aggregate adhesion of the binder system. At modifier contents of 0%, 3%, 6%, 9%, 12%, and 15%, the pull-off strengths of the WEA binders are 1.27 MPa, 1.63 MPa, 1.81 MPa, 2.03 MPa, 2.29 MPa, and 2.62 MPa, respectively. However, WER increases the low-temperature stiffness modulus of WEA binders and reduces the creep rate, thereby adversely affecting the low-temperature flexibility of the system.

This work revealed the microscopic interaction mechanism between WER and emulsified asphalt and demonstrated that the WER significantly enhanced the high-temperature rutting resistance and adhesion of emulsified asphalt. However, the dense cross-linked network formed after WER curing adversely affects the low-temperature performance of the binder. Research on the physical toughening or chemical toughening of the system represents a crucial direction for expanding the applicability of the WEA binders. Furthermore, as WEA maintenance materials are applied to pavement surface layers, they are subjected to the coupled effects of traffic loading and environmental factors. Consequently, the durability of the WEA binder is also a critical factor influencing the performance of the maintenance materials. Future investigations into the aging mechanisms and performance degradation processes of WEA binders and their derived maintenance materials under complex environmental conditions are of significant importance.

## Figures and Tables

**Table 1:** Non-ionic WER emulsion.

Ionic Type	Epoxy Value	Viscosity (25 °C, mPa·s)	pH	Solid Content (%)	Average Particle Size (μm)
Non-ionic	0.23	800	8	55	1.5

**Table 2:** Modified-amine curing agent.

Appearance	Active Hydrogen Equivalent	Solid Content (%)	pH	Viscosity (25 °C, mPa·s)	Specific Gravity
Light yellow uniform fluid	240	50.5	9.5	600	1.04

**Table 3:** Neat emulsified asphalt.

Demulsification Speed	Angler Viscosity, 25 ℃	Solid Content (%)	Penetration (25 °C, 0.1 mm)	Ductility (15 °C, cm)	Softening Point (°C)	Storage Stability (%)
Slow setting	3.7	60	84	≥100	46.8	0.8

**Table 4:** Three types of liquid SFE parameters.

Liquid	SFE (*γ*, mJ/m^2^)	Dispersion Component (*γ^LW^*, mJ/m^2^)	Polar Component (*γ^AB^*, mJ/m^2^)
Formamide	59.0	39.4	19.6
Glycerol	65.2	28.3	36.9
Distilled water	72.3	18.7	53.6

**Table 5:** Main absorption peaks in infrared spectra.

Absorption Peaks/cm^−1^	Functional Groups	Vibration Form
3395, 3346, 3366	-OH, -NH	Stretching vibration
2851–2963	C-H in alkyl groups	Symmetric and antisymmetric stretching vibrations
1739	-C=O	Stretching vibration
1607, 1508	-C=C- from benzene ring	Stretching vibration
1456	C-H	In-plane stretching vibration
1376	-CH3	Bending vibration
1034–1298	C-O bond at different positions	In-plane stretching vibration
721–874	C-H on benzene ring	Out of plane rocking vibration

**Table 6:** Contact angle (*θ*) of WEA binders with different modifier content.

WER Content (%)	Distilled Water		Glycerol		Formamide	
	Average Value (°)	C.V. (%)	Average Value (°)	C.V. (%)	Average Value (°)	C.V. (%)
0	98.3	0.89	90.0	0.22	83.4	0.66
3	95.1	0.96	87.2	0.45	81.2	0.23
6	93.6	0.09	86.1	0.58	80.5	0.08
9	91.3	182	84.1	0.68	76.8	0.24
12	88.3	0.93	82.3	0.41	74.2	1.21
15	86.7	1.01	80.8	0.79	73.1	0.81

**Table 7:** SFE parameters of WEA binders.

WER Content (%)	*γ* (mJ/m^2^)	*γ^LW^* (mJ/m^2^)	*γ^AB^* (mJ/m^2^)
0%	18.09	14.74	3.35
3%	19.13	14.59	4.54
6%	19.40	14.06	5.34
9%	21.3	15.68	5.62
12%	22.41	15.44	6.97
15%	23.13	15.29	7.84

## Data Availability

The original contributions presented in this study are included in the article. Further inquiries can be directed to the corresponding author.
